# Thermochemistry of Sulfur-Based Vulcanization and of Devulcanized and Recycled Natural Rubber Compounds

**DOI:** 10.3390/ijms24032623

**Published:** 2023-01-30

**Authors:** Franco Cataldo

**Affiliations:** Actinium Chemical Research, Via Casilina 1626A, 00133 Rome, Italy; franco.cataldo@fastwebnet.it

**Keywords:** bonding between adjacent polymer chains, sulfur-based crosslinks, DSC, thermochemistry, vulcanization, mechanochemistry, mechanophore, devulcanization, reclaim, recycling

## Abstract

The vulcanization of rubber compounds is an exothermal process. A carbon black-filled and natural rubber-based (NR) formulation was mixed with different levels of sulfur (0.5, 1.0, 2.0, 4.0 and 6.0 phr) and studied with differential scanning calorimetry (DSC) for the determination of the vulcanization enthalpy. It was found that the vulcanization enthalpy is dependent on the amount of sulfur present in the compound and the vulcanization heat released was −18.4 kJ/mol S if referred to the entire rubber compound formulation or −46.0 kJ/mol S if the heat released is referred only to the NR present in the compound. The activation energy for the vulcanization of the rubber compounds was also determined by a DSC study at 49 kJ/mol and found to be quite independent from the sulfur content of the compounds under study. A simplified thermochemical model is proposed to explain the main reactions occurring during the vulcanization. The model correctly predicts that the vulcanization is an exothermal process although it gives an overestimation of the vulcanization enthalpy (which is larger for the EV vulcanization package and smaller for the conventional vulcanization system). If the devulcanization is conducted mechanochemically in order to break selectively the sulfur-based crosslinks, then the natural rubber compounds recovered from used tires can be re-vulcanized again and the exothermicity of such process can be measured satisfactorily with DSC analysis. This paper not only proposes a simplified mechanism of vulcanization and devulcanization but also proposes an analytical method to check the devulcanization status of the recycled rubber compound in order to distinguish truly devulcanized rubber from reclaimed rubber.

## 1. Introduction

Vulcanization is a chemical reaction which was discovered in the first half of the 19th century and involves a reaction between elemental sulfur and diene rubbers in general. More specifically, elemental sulfur (under the form of elemental sulfur cyclooctasulfur and more rarely polymeric sulfur known also as insoluble sulfur) is dissolved and/or dispersed in a rubber matrix (together with other compounding ingredients) and then subjected to a heat treatment. As a result of this thermal treatment, the rubber compound passes from a plastic state to a final stable and elastic state with a definitive form. The result of this chemical transformation is explained by the formation of sulfur bonds or “bridges” connecting adjacent rubber chains which are no more free to flow completely with respect to each other (as was possible before vulcanization). The sulfur crosslinks permit only a restricted flow of the rubber chain segments and acts as return springs leading to the desired viscoelastic response typical of the rubber vulcanizates. 

The incredible impact on modern life derived from this ancient chemical reaction is widely recognized, with the creation of a number of technical articles and rubber composites of various form and functions.

The process of sulfur-based vulcanization chemistry has been reviewed several times in the last 50 years [1,2,3,4,5,6,7,8,9]. From the extreme complexity of the chemical reactions involved, some milestones have been identified. First of all, the free radical mechanism has been definitely clarified as the key mechanism involved in S-based vulcanization (especially under unaccelerated conditions), although other parallel mechanisms (e.g., polar ion-radical) may by advocated in some accelerated curing circumstances [1,2,3,4,5,6,7,8,9]. The other milestone regards the reaction sites along the diene rubber chains where the sulfur bonds are attached to form the “bridges” connecting the adjacent rubber chains. Such reaction sites are definitely located at the allylic position to the double bonds [1,2,3,4,5,6,7,8,9]. Thus, in the case of natural rubber or cis-1,4-polyisoprene which is among the subjects of this paper (since it is extracted from the renewable sources primarily from *Hevea brasiliensis* or–seldom-from other plants such as, for instance, *Parthenium argentatum*), the allylic positions to the double bonds are represented by the three sites in α to the double bond of each isoprene unit of the polymer chain. The point is that the C-H bond dissociation energy (DBE) at the allylic position to a double bond is particularly low with respect to other C-H BDE and is particularly targeted by the hydrogen free radical abstraction reaction [10,11]. The resulting allyl radical is stabilized by conjugation with the adjacent double bond and remains long-lived enough to be saturated by an incoming mono- or polysulfur radical [1,2,3,4,5,6,7,8,9]. Another milestone reached by the vulcanization studies regards the distribution of mono-, di-, and polysulfidic crosslinks. It has been established that in the case of unaccelerated compounds or compounds formulated with conventional vulcanization system (i.e., excess of sulfur over the accelerator), eventually with low zinc content and relatively low curing temperatures with prolonged times, the rubber network will be governed by the presence of an excess of polysulfidic crosslinks, minimal presence of disulfidic bridges and absence of monosulfidic crosslinks [1,2,3,4,5,6,7,8,9]. Conversely, the use of EV systems in the rubber compound formulations (characterized by a prevalence of accelerator over the sulfur content) with adequate ZnO and stearic acid content, ensures the absolute prevalence of monosulfidic crosslinks over the disulfidic with the complete absence of polysulfidic bonds. Furthermore, these results are achieved with relatively short curing times at relatively high temperature conditions [1,2,3,4,5,6,7,8,9]. Another feature of the vulcanization was the discovery made at the National Bureau of Standards (NBS) in 1969 that the vulcanization reaction is an exothermal reaction [12,13]. In fact, until the careful work made by the NBS scientists, it was not clear that sulfur vulcanization was an overall exothermic reaction at any sulfur loading levels. It was instead thought that the exothermicity of the vulcanization reaction was observable only in natural rubber compounds containing sulfur above the 5% level threshold [13]. It was instead demonstrated [12] and confirmed later that the exothermicity of sulfur reaction with natural rubber occurs at any sulfur content level, even in normal rubber compounds formulations [13,14,15].

The long road toward the maturation of our knowledge of the complexity involved in the S-based vulcanization process, has led to impressive technological achievements with rubber products having excellent durability, mechanical resistance and fine-tuned viscoelastic properties. 

The great steps ahead and the strong efforts in our understanding of the vulcanization chemistry were not applied in the same way also on the rubber recycling processes and in our understanding of the devulcanization of used rubber products. In fact, the current and most common recycling processes of used rubber compounds are synonym of reclaiming processes and the conventional reclaiming processes were invented in the first half of the XX century [16]. These processes involve the indiscriminate administration to the recycled rubber of heat, pressure, mechanical shear, use of chemical additives, reagents [17,18,19,20] and softeners to produce at the end of a long process, a refined reclaim but which cannot be defined “devulcanized rubber” but reclaimed rubber [16,20]. In contrast, a really devulcanized rubber compound should be conceived as a formerly cured and used rubber compound which has undergone a selective mechanochemical processing targeted exclusively to break the sulfur-based crosslinks without causing the undesired main rubber chain scission as well as avoiding generalized mechanochemical oxidative degradation. These concepts were perhaps first expressed more than twenty years ago [20,21,22], were expanded by Myhre and Khait in their contribution to the book “Rubber Recycling” [23,24] and then discussed and expanded in a clearer and distinct way in a series of excellent review works specifically dedicated to devulcanization [24,25,26,27,28,29,30,31,32,33,34,35].

In the present work, we will start with the principle that vulcanization is an exothermal reaction process, we will study such exothermal reaction in a carbon black filled NR-based compound at different sulfur loadings and we will examine in a simplified model the molecular reasons linked to such exothermicity. Then, we will apply these concepts to the recycled rubber compounds. Conventionally recycled rubber compounds or reclaims, once re-heated do not show any exothermal transition. Conversely, true devulcanized rubber compounds once heated should show evidence of an exothermal transition due to re-vulcanization. Consequently, the differential scanning calorimetry (DSC) could be used as a tool to differentiate conventional rubber reclaims from truly devulcanized recycled rubber.

## 2. A Lesson on the Vulcanization Enthalpy of NR/BR Compound

**Figure 1:**
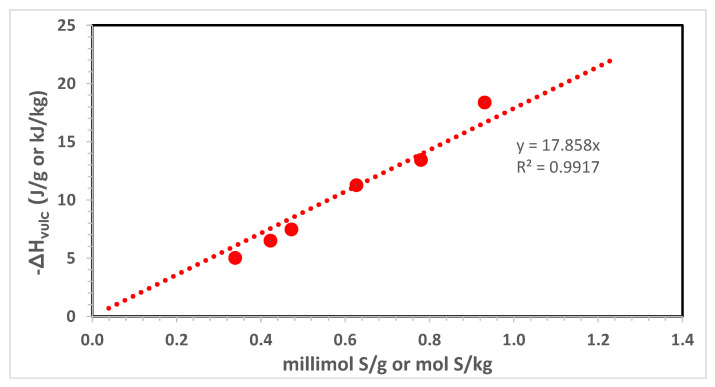
Vulcanization enthalpy measured with DSC on the following formulation: NR 80 phr; BR 20 phr; N326 carbon black 50 phr; paraffinic plasticizer oil 17 phr; stearic acid 3 phr; zinc oxide 3 phr; antioxidant TMQ 3 phr; accelerator NMOR 1.2 phr; sulfur variable: 1.2; 1.5; 1.68; 2.24; 2.8; 3.36 phr. All data taken from ref. [13] and re-elaborated in the present work.

The main lesson learned from the workers from the National Bureau of Standards (NBS) [12,13,14,15] regarding the vulcanization enthalpy of the sulfur-based natural rubber/polybutadiene (NR/BR) compounds concerns the fact that the heat evolved during the vulcanization process is directly proportional to the amount of sulfur present in the rubber compound. In other words, the entity of the exotherm it depends from the amount of sulfur which remained linked to the isoprene units of the rubber chains or, if preferred, by the reaction of the allylic sites of the isoprene units with sulfur diradicals under the form of mono-, di- and polysulfide diradicals. This trend can be observed in Figure 1 using the experimental data taken from ref. [13] expressed in J/g of vulcanization heat and reporting the amount of sulfur in millimol of sulfur per g of compound, the slope results in −17.9 J/millimol S which is equivalent to −17.9 kJ/mol S. This value is referred to as the heat released by the entire rubber compound. If we want to refer the vulcanization heat released only to the rubber matrix, considering that it represents about 40% by mass, then the vulcanization heat is recalculated as −44.7 kJ/mol S. 

Other interesting results derived from the work of the NBS scientists regards the fact that the vulcanization enthalpy is independent from the chemical nature of the accelerator and dependent only to the amount of sulfur present in the rubber compound [12,13,14,15]. The role of the accelerator is to reduce the onset and the peak temperature of the vulcanization reaction with respect to an analogous unaccelerated compound [12,13,14,15]. Another important aspect clarified by NBS scientists regards the fact that variable amounts of accelerator do not affect the vulcanization heat whose value remains constant and dependent almost exclusively from the amount of sulfur present [12,13,14,15].

## 3. Measurement of the Vulcanization Enthalpy in a NR-Based Compound

The research work on the vulcanization enthalpy at NBS was carried out essentially on a NR/BR (80/20 phr) rubber blend, and it was shown that the nature of the diene polymers involved in the vulcanization affects the vulcanization exotherm and in general the presence of BR and/or SBR in the rubber compound leads to a significant increase of the heat released with respect to a reference NR compound [13]. Thus, in the present study, we have selected a conventional and NR-based formulation which was declined in 5 different S levels as shown in Table 1. Our focus on NR derives from the fact that cis-1,4-polyisoprene is extracted from plants and represents a completely renewable source and it is used in tires application as well as in different types of rubber goods and technical articles. As shown in Table 1, all components of the formulation were kept constant and only the sulfur level was increased progressively from compound A to compound E. It is worth noting here that a vulcanization system where the amount of accelerator is prevalent over the amount of sulfur is called EV (efficient vulcanization system) and compound A and B in Table 1 are in line with an EV system. When the amount of accelerator and sulfur are approximately at the same level, it is talked about semi-EV vulcanization system and compound C in Table 1 may be considered in line with such system. Finally, when the amount of sulfur is prevalent above the accelerator, the vulcanization system is defined as conventional and compounds D and E in Table 1 are matching such a condition. 

As explained in different treatises or monographs on vulcanization [1,2,3,4,5,6,7,8,9], the substantial difference between the three mentioned vulcanization systems regards the nature and distribution of the sulfur bridges bonding adjacent rubber chains. With the EV system the sulfur bridges are by far monosulfidic and seldom disulfidic. With the semi-EV vulcanization system, the formation of monosulfidic bridges are suppressed in favor of the disulfidic and polysulfidic. Finallly, in the case of conventional cure system, the polysulfidic bridges are the most common followed seldom by the disulfidic while the monosulfidic are completely absent. Thus, with our five rubber compounds in Table 1 we are covering all of the possible vulcanization systems. 

**Figure 2:**
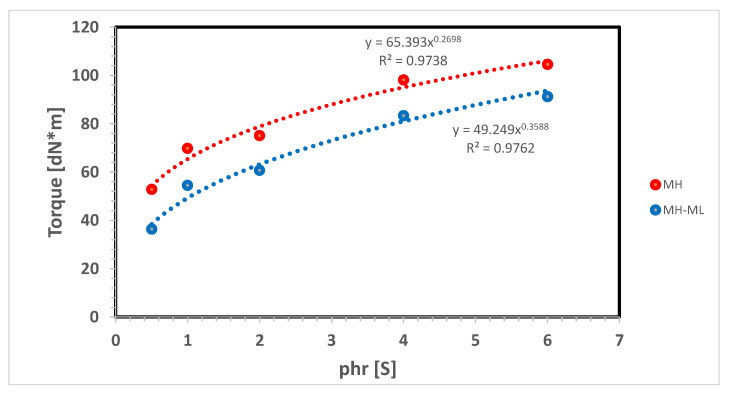
Maximum rheometer torque (MH) and delta torque (MH-ML) measured on the five compounds of Table 1. The rheometer test was run for 8 min at 175 °C. The torque values grow following the amount of sulfur added, implying a higher crosslinking density.

As shown in Figure 2, all of the rubber compounds in Table 1 as soon as prepared were checked with a conventional oscillating disk rheometer (ODR) test to assess that they were correctly prepared with the scheduled amount of sulfur. In Figure 2 the maximum torque values (MH) reached by each rheometer curve made on each rubber compound of Table 1 are reported together with the delta torque values (MH-ML) which represent an indirect indication of the crosslinking density reached by each compound under study. From Figure 2 it is immediately evident that both MH and MH-ML increase following the sulfur content of each compound, since it is the amount of sulfur which governs the stiffness and the crosslinking density of the resulting cured rubber compound. The linear fitting of the experimental data is already satisfactory, but, as shown in Figure 2, the fitting with a power law gives an even better R^2^ value than linear fitting. Thus, the following power law was preferred:

MH = 65.393 [phr S]^0.2698^ (1)

and

MH-ML = 49.249 [phr S]^0.3588^ (2)

**Figure 3:**
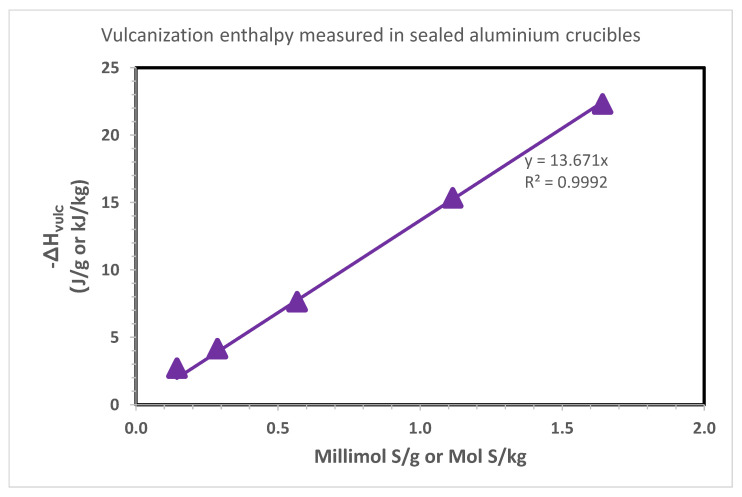
Vulcanization enthalpy measured with DSC on the compounds of Table 1. Heating rate 10 °C/min in sealed Al crucibles from room temperature to 350 °C. The vulcanization thermal parameters are shown in Table 2.

The first DSC study on the compounds in Table 1 was performed using conventional Al crucibles. However, the crucibles were loaded with the compounds and sealed with the Al cap without any conventional punch at the top. The DSC scan was made at 10 °C/min from room temperature to 350 °C. Table 2 summarizes the onset and peak temperatures for the vulcanization exotherms of all of the samples analyzed while Figure 3 shows the correlation between the vulcanization enthalpy and the sulfur content (expressed in mmol/g compound). From Table 2, it is evident that the onset of the vulcanization process is dependent from the sulfur content of the compounds. Higher sulfur content requires a higher onset temperature. This phenomenon is linked to the necessity to break the molecules of cyclooctasulfur into smaller sulfur chains diradicals (which are the active sulfurating species) and higher S content requires higher temperatures. Instead, the vulcanization peak is always found at about 180 °C with the exception of the compound with the maximum sulfur content whereas the peak is shifted slightly above 200 °C.

Figure 3 shows a good linearity in terms of vulcanization heat released as function of the sulfur content of the NR-based compounds. From the slope of the line Figure 3 the vulcanization enthalpy is found at −13.7 J/mmol S or, which is the same kJ/mol S. This enthalpy value is referred to the complete rubber compound formulation. Since the compounds in Table 1 contain about 40% by mass or rubber matrix, then the vulcanization enthalpy can be re-calculated at −34.2 kJ/mol S per unit mass of NR.

The vulcanization enthalpy measured on the NR compounds of Table 1 results about 76.5% the value reported by the NBS scientists. 

However, the Al crucible, although sealed, may not necessarily withstand the pressures that develop during vulcanization (with the release of some volatile matter leading to heat loss). Of course, this phenomenon may lead to an underestimation of the vulcanization enthalpy. 

To overcome these drawbacks presented by conventional Al crucibles, 30 μL stainless steel medium pressure crucibles from Mettler-Toledo (code 51140404) were selected for the determination of the vulcanization enthalpy. These crucibles are re-openable and permit to accommodate more than 20 mg of rubber compound per DSC scan.

**Figure 4:**
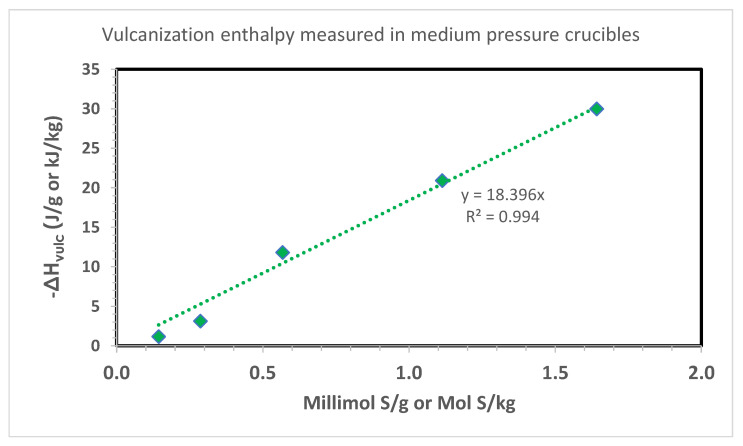
Vulcanization enthalpy measured with DSC on the compounds of Table 1. Heating rate 20 °C/min in stainless steel medium pressure crucibles from room temperature to 350 °C. The vulcanization thermal parameters are shown in Table 3.

The results of this study in a medium pressure crucible are shown in Table 3 whereas a heating rate of 20 °C/min was selected instead of 10 °C/min of the tests made in the Al crucibles. Table 3 shows that in these conditions the onset temperature for the vulcanization process is found around 180 °C, at significantly higher temperature than the study with Al crucibles. This is reflected also on the vulcanization peak temperature which can be found around 200 °C in Table 3 for the study in medium pressure crucibles with respect to the Al crucibles (Table 2). In general, the high onset and peak temperature observed in Table 3 for the vulcanization study with medium pressure crucibles can be attributed first of all to the higher heating rate which tends to shift the thermal transition to higher temperatures, but also to the higher mass and thermal inertia of the stainless steel crucibles with respect to the Al crucible and the higher rubber compound mass confined inside the crucibles. In terms of vulcanization enthalpy, Table 3 shows in general higher values than Table 2. These data are plotted in Figure 4 and show a good linearity between the vulcanization enthalpy and the amount of sulfur present in the compounds under study (Table 1). This time the slope of the line in Figure 4 suggests that the vulcanization enthalpy is −18.4 J/mmol S or kJ/mol S, a value much higher than that determined in Figure 3 and in fair agreement with the vulcanization enthalpy determined by the NBS scientists (Figure 1), i.e., −17.9 kJ/mol S. Thus, for the determination of the vulcanization enthalpy the stainless steel medium pressure crucibles are certainly more suitable the than the Al crucibles which cannot withstand adequately the high pressure generated by the vulcanization process. As usual, the value of −18.4 kJ/mol S determined in Figure 4 is referred to the entire rubber compound formulation. Since the rubber content represents about 40% of the total mass, if the vulcanization enthalpy is multiplied by a factor 2.5 it gives the vulcanization enthalpy referred only to the NR content (i.e., −46.0 kJ/mol S), again in fair agreement with the value of −44.7 kJ/mol S determined by the NBS scientists (see Figure 1 and related discussion).

## 4. About the Activation Energy for the Vulcanization

It was reported that the Arrhenius activation energy for the unaccelerated vulcanization (thus, direct reaction of sulfur with diene rubber sites) is comprised between 138 and 150 kJ/mol [1]. The last figure was confirmed in a more recent review on the properties of liquid sulfur [36] whereas the homolytic scission of the S-S bond in cyclooctasulfur indeed involves an enthalpy of 150 kJ/mol as measured by electron spin resonance analytical technique. However, Tobolsky and Eisenberg in their equilibrium model of liquid sulfur have obtained a ΔH_r_ = +137 kJ/mol for the reaction S_8_(cycle) → S_8_(chain) [36] which is in the lower limit of the activation energy reported by Porter for the Arrhenius activation energy of vulcanization. 

The initial heating of a rubber compound has a precise scope to start the vulcanization reaction and to overcome the initial step of the activation energy for the vulcanization. The energy-demanding step is indeed the scission of cyclooctasulfur ring in order to produce the sulfur free radicals which are involved in the vulcanization. In the presence of accelerators and activators (ZnO and stearic acid) the activation energy involved in this step is lowered considerably from the values reported above for unaccelerated rubber compound.

In fact, we have measured by DSC the activation energy for the vulcanization of compound B (Table 1) which has an EV (efficient) cure system in comparison to compound E (Table 1) which instead has a conventional vulcanization system with a large excess of sulfur over the accelerator. The rubber compound samples were heated in high pressure crucibles at different heating rates ranging from 5 °C/min to 60 °C/min and the onset and peak vulcanization temperatures were determined for each compound. Then, the experimental data were treated according to the Ozawa and Kissinger methods which are widely used for the evaluation of the activation energy of a chemical reaction studied at the DSC [37,38,39,40,41]. The Ozawa equation is the following [42]:

(2.15Logβ) = (−E^#^/R) (10^3^/T_peak_) (3)

while the Kissinger equation is [42]:

[2.303Log(β/T^2^)] = (−E^#^/R) (10^3^/T_peak_) (4)

In these equations β represents the heating rate, T is the peak temperature of the thermal phenomenon studied and R is the universal gas constant. The two equations give in general very similar results with comparable accuracy [42].

**Figure 5:**
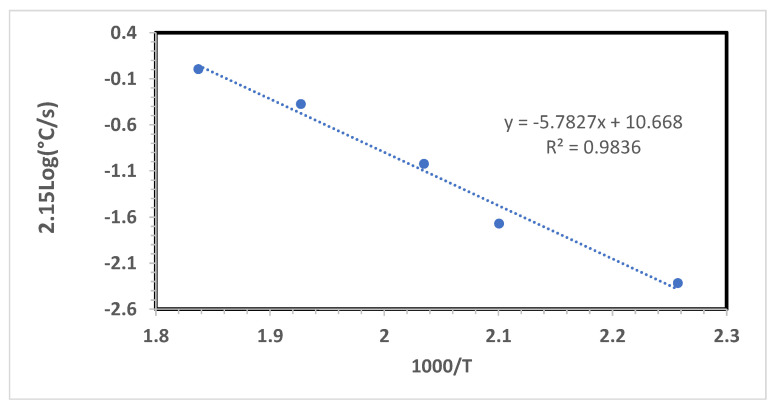
Determination of the activation energy for the vulcanization on compound B, Table 1 according to the Ozawa equation. The activation energy is represented by the slope which, multiplied by the gas constant R it gives an E^#^ = 48.0 kJ/mol. The Kissinger method gives a comparable activation energy of E^#^ = 47.0 kJ/mol.

In Figure 5 are shown the experimental results regarding the compound B of Table 1 having an EV cure package. Five curing rates were used, namely 5 °C/min, 10 °C/min, 20 °C/min, 40 °C/min, 60 °C/min and from the slope of the graph of Figure 5 an activation energy E^#^ = 48.0 kJ/mol, following the Ozawa equation 81). The Kissinger Equation (2) in a similar mathematical treatment has given a practically analogous E^#^ = 47.0 kJ/mol.

**Figure 6:**
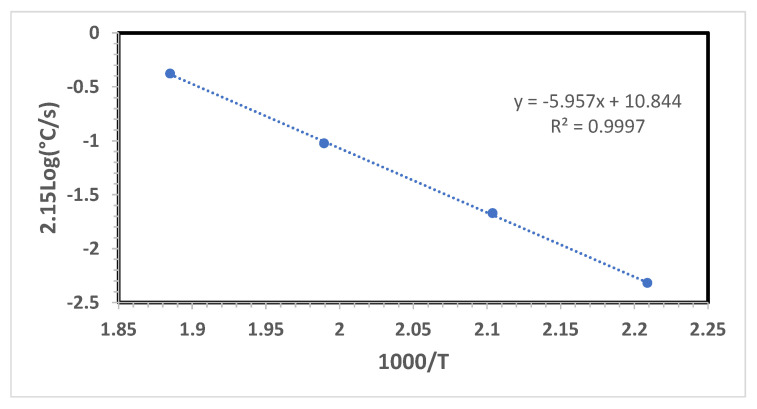
Determination of the activation energy for the vulcanization on compound E, Table 1 according to the Ozawa equation. The activation energy is represented by the slope which, multiplied by the gas constant R it gives an E^#^ = 49.5 kJ/mol. The Kissinger method gives a comparable activation energy of E^#^ = 49.0 kJ/mol.

Thus, the experimental activation energy was found about 1/3 the value expected for the unaccelerated vulcanization (i.e., 137–150 kJ/mol), as discussed previously. Surprisingly, compound E from Table 1 which is characterized by a conventional cure package shows just a slightly higher activation energy for vulcanization than compound B. In fact, in Figure 6 are reported the results of the DSC measurements made on compound E (Table 1) and the resulting E^#^ derived according to the Ozawa Equation (1) was found at 49.5 kJ/mol, substantially confirmed by the Kissinger Equation (2) with an E^#^ = 49.0 kJ/mol. Evidently, the presence of an accelerator, irrespective for the sulfur content of the given rubber compound, leads inevitably to a dramatic reduction of the activation energy for the vulcanization to approximately 1/3 the value expected for unaccelerated vulcanization. Indeed, the role of the accelerator is to break down the cyclooctasulfur molecule into sulfur free radical fragments (e.g., •S-S-S-S•; •S-S-S-S-S-S•; •S-S•; etc…) which can be considered the effective sulfurating species of the rubber matrix. In the absence of the accelerator, the cyclooctasulfur breakdown is instead a purely thermal reaction and hence not only it is slow and inefficient, it requires prolonged heating and a high value of the activation energy. Of course, the vulcanization mechanism is much more complicated by this just given simplified view and not only the accelerator but also ionic zinc plays a key role in the formation of the active sulfurating species effective in the formation of the crosslinks in the rubber matrix. For these details, it is certainly necessary to read the specific literature [1,2,3,4,5,6,7,8,9] on the vulcanization mechanism. However, in the following discussion, we will adopt a simplified model of vulcanization thermochemistry (without involving zinc complexes sulfurating species) which matches reasonably well the experimental results.

## 5. Vulcanization Thermochemistry: A Simplified Model

In Table 4 are collected a series of reaction enthalpies involving the molecular sulfur (cyclooctasulfur) breakdown into sulfur radicals. The reaction enthalpies were calculated either using the sulfur-centered free radical data from ref. [43] or using the Benson’s data taken from ref. [44].

From the data in Table 4, it is immediately evident that the lowest reaction enthalpy is the ring opening of cyclooctasulfur through the homolysis of one S-S bond with the production of a chain of the type •S-S-S-S-S-S-S-S• [36]. From the calculated enthapies of reaction, also the production of •S_7_•, •S_6_• and •S_5_• appear more favored than shorter sulfur chains that in their turn require more energy. In other words, all of the reactions considered in Table 4 are endothermic reactions which, depending on the external energy available, are activated first by the reactions requiring lower enthalpy than those requiring larger enthalpy values. The precise role of the accelerator and activator (ZnO) are to facilitate the cyclooctasulfur ring breakdown, to shorten the radical sulfur chain, and to build a complex with zinc and accelerator components as ligands acting as active sulfurating species [1,2,3,4,5,6,7,8,9]. The energetics of this very step with zinc complex as the sulfurating specie is too complex to be calculated with any degree of accuracy. From the data in Table 4 it is evident that a theoretical activation energy threshold at 137–150 kJ/mol is fully justified while the actual experimental values measured on a couple of compounds of Table 1 and found at about 49 kJ/mol is explainable by the effect of accelerators and activators present in the rubber compounds.

**Scheme 1:**
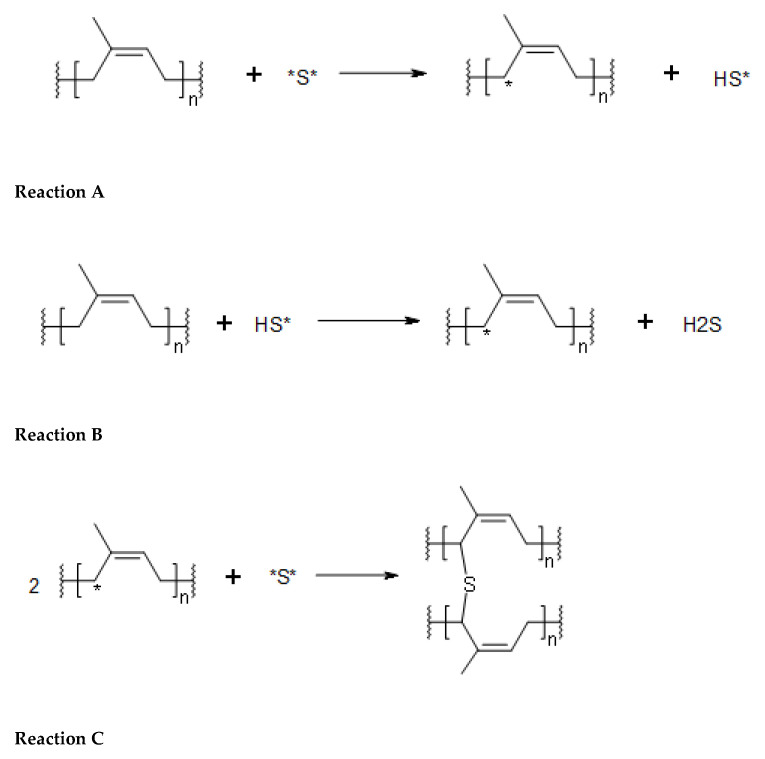

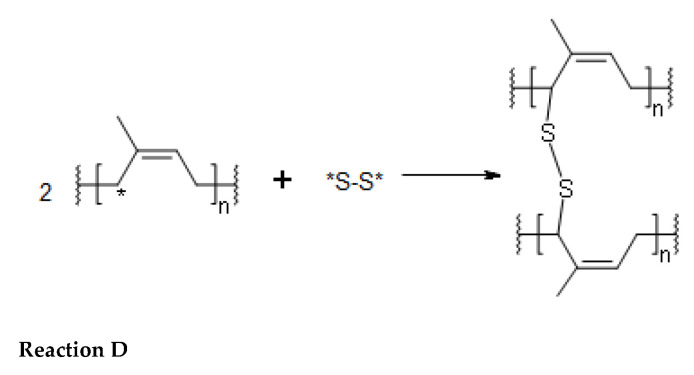
The reactions **A**,**B**,**C**,**D** leading to crosslinks between isoprene units sulfur diradical species connecting adjacent rubber chains with sulfur bridges crosslinks. (**Reaction A**) Monosufide diradical abstracts a hydrogen atom at the allylic position of the isoprene unit forming a monohydrosulfide radical; (**Reaction B**) The monohydrosulfide radical coming from reaction A abstracts another H atom at the allylic position of another isoprene unit, producing hydrogen sulfide; (**Reaction C**) The monosulfide diradical combines with two allylic sites of isoprene units from adjacent rubber chains forming a monosulfidic crosslink. (**Reaction D**) A Disulfide diradical combines with two allylic sites of isoprene units from adjacent rubber chains forming a disulfidic crosslink and the same reaction can be extended to polysulfidic bridges with 3,4,5,….sulfur chains.

The next step of our simplified model regards the formation of the allyl radical in a polyisoprene chain. Scheme 1 details the steps involved. In our simplified model the allyl radical in certain isoprene units are generated by hydrogen abstraction reaction of the allylic hydrogen caused by the sulfur diradicals of any length. The resulting monohydrogensulfide or polysulfide radical then abstracts another allylic hydrogen atom in another isoprene unit sites. The final step is the sulfurization of the allylic sites. 

To make some thermochemical calculations, the enthalpy of formation of the allyl radical in certain isoprene unit was taken as 92.0 kJ/mol since such value is tabulated for the case of 2-pentene-4-radical and 3-methyl-2-butene-4-radical [10,11]. The latter two structures resemble the isoprene monomeric unit and are characterized by the presence of an allyl radical conjugated with a double bond. Once known the enthalpy of formation of the allyl radical in the polyisoprene units, we need to know the enthalpy of formation of the pristine isoprene unit and the enthalpy of formation of the crosslinked adjacent rubber chains through the sulfur bridges of any length. These enthalpies of formation were calculated using the group increment approach proposed by Van Krevelen [45]. Essentially, Δ_f_H° = −2.0 kJ/mol was calculated for the isoprene monomeric unit, Δ_f_H° = +74.6 kJ/mol for two isoprene monomeric units crosslinked by a monosulfide bridge, +80.6 kJ/mol if crosslinked by a disulfide bridge, +84.1 kJ/mol for a trisulfide bridge and +87.5 for a tetrasulfide bridge. The enthalpy of formation of all sulfur diradicals or monohydrogensulfide and monohydorgenpolysulfide radicals were taken primarily from the ref. [43] and alternatively from [44] although very little differences were found in the enthalpies of formation from the two mentioned literature sources for each molecular (or radical) specie considered. With all of these data available, the enthalpy of reactions from Reaction A to reaction D in Scheme 1 were calculated and reported in Table 5.

From the reaction enthalpies reported in Table 5, it is evident that Reaction A and B for the formation of the allylic sites on the isoprene units are definitely exothermic if the reagent is the monosulfur diradical and appear slightly endothermic in the case of the action of disulfide or polysulfide diradicals. However, the reactions C–D of radical recombination whereas the allylic sites of the isoprene units combine with sulfur or polysulfur diradicals to produce the crosslinks of any length are largely exothermic and shorter chains sulfur crosslinks seem favored from the enthalpy change in comparison to longer chains. The last row at the bottom of Table 5 gives the enthalpy sum of all of the reactions shown in Scheme 1 and considered in the current simplified model showing that in all cases the reactions taken together give an exothermal response as indeed observed experimentally. 

Now, in an EV vulcanization system it is well known that there is a large prevalence of monosulfidic crosslinks accompanied by a minor amount of disulfidic bonds [1,2,3,4,5,6,7,8]. Assuming somewhat arbitrarily a ratio 80/20 monosulfidic/disulfidic in an EV system and considering negligible the polysulfidic contribution, then the theoretical enthalpy of reaction is [0.8 × (−520.2) + 0.2 × (−141.2)] × [0.2 (mol S)] = −88.9 kJ (using the data at the bottom of Table 5 and the mol of S of Table 1 compound A or B) which is about 9.5 times the value experimentally measured of −46 × 0.2 = −9.2 kJ. Instead, in the case of semi-EV vulcanization system where the polysulfide crosslinks play a role, we may assume a ratio di/tri/tetrasulfide = 40/30/30 and in this instance the vulcanization enthalpy may be estimated as follows: [0.4 × (−141.2) + 0.3 × (−115.9) + 0.3 × (−93.9)] × [0.57 mol S] = −68.1 kJ (using again the data at the bottom of Table 5 and the mol of S of Table 1 compound C). The latter value is just 2.5 times the experimental value of −46 × 0.57 = −26.2 kJ/mol. In the limiting case of compound D of Table 1 where the S content is 1.11 mol/kg, the vulcanization package is of conventional type. Assuming an exclusive polysulfidic content, the vulcanization enthalpy is calculated as follows: [(1 × (−93.9)] × [1.11 mol S] = −104.2 kJ which is just 2 times the actual experimental value of −46 × 1.11 = −51.1 kJ. Applying the same reasoning to compound E of Table 1 which is characterized by a conventional cure package and its S content is 1.64 mol/kg, the theoretical calculation gives [(1 × (−93.9)] × [1.64 mol S] = −154.0 kJ which is just 2 times the actual experimental value of −46 × 1.64 = −75.4 kJ.

In other words, as soon as we pass from the EV vulcanization system to the semi-EV vulcanization system and the to the conventional system the gap between the experimental value and the theoretical values is reduced significantly. This means that the simplified model for the calculation of the vulcanization enthalpy matches more effectively the conventional vulcanization system rather than the EV and semi-EV system. This is expected, since the simplified calculation model does not consider at all of the role of accelerator and activator and the conventional system (such as compound E in Table 1) is so rich of sulfur that it is closer to the simplified vulcanization model used.

Thus, if the simplified model works, the effect of the accelerator and activator at the end is to reduce the vulcanization exotherm with respect to an unaccelerated rubber compound.

In summary, the activation energy for the vulcanization in our rubber compounds was found around 50 kJ/mol (measured as activation energy for the vulcanization) and corresponds to the steps considered in Table 4 (i.e., cyclooctasulfur molecule breakdown into radical chains, an endothermal step). Theoretically this value is instead expected at about 150 kJ/mol for not accelerated rubber compound. Thus, the presence of accelerator and activator reduce the activation energy for the vulcanization. On the other hand, the vulcanization enthalpy is an exothermal process releasing 46 kJ/mol S as measured in our compounds of Table 1 and the theoretical calculation of our simplified vulcanization model confirms the exothermicity of the vulcanization reaction with an overestimation of the heat released which is larger for the compounds with EV and semi-EV vulcanization systems and smaller for the compounds with conventional vulcanization systems which are those more closer to the simplified model used.

Other aspects which may be rationalized with the simplified vulcanization model and the calculations of Table 5 regard a series of phenomena occurring after the vulcanization. These phenomena are known as the sulfur-sulfur bond interchange, desulfurization and resulfurization reactions [3]. Without entering into the complex details of these phenomena (which was analyzed in detail for example in [3]), we may say that the sulfur network in a vulcanizate is not stable, especially if it is rich of polysulfide and disulfide bridges. Table 6 provides a series of bond dissociation energies (BDE) of chemical bonds involved in vulcanized network. It is immediately evident that polysulfidic chains represent the weaker sites in the network and for a number of reasons (action of heat, mechanical stress-strain, mechanical heat-build-up, mechanical compression, etc…) are subjected to homolytic breakdown and extrusion of sulfur, so that the vulcanized network shows a tendency to rearrange preferably into a more stable network with less possible polysulfidic bonds. During these rearrangements there is also an increase in the crosslinking density and, of course, a tendency toward mono- and disulfidic crosslinks. However, this trend toward a more stable network finds an immediate explanation in the thermodynamics calculation of Table 5 whereas a monosulfidic crosslinked network represent the most stable state with the lowest enthalpy of formation value, followed by far by the disulfidic network and then trisulfidic and tetrasulfidic which are not far from the disulfidic from the enthalpy of formation standpoint.

## 6. Devulcanization and DSC Measurement on Devulcanized Rubber Compounds

As accounted by Casale et al. [46] and more briefly by Brydson [2], the concept of mechanochemistry was created out of the mastication of the natural rubber (NR) at the beginning of the history of polymer science. As is well known to the experts in this art, natural rubber supplied to the rubber mixing plants is delivered with a quite high molecular weight and then it should be adequately milled to reduce its molecular weight to improve its workability and to allow an easier incorporation of the fillers and the other compounding ingredients. To facilitate such milling operation to reduce its original molecular weight, free radical sources (in general thiophenol derivatives or compounds) are added in very small quantities [2,46].

**Table 6:** Bond dissociation energy (BDE) of chemical bonds involved in vulcanized networks.

	BDE (kJ/mol)	BDE (kJ/mol)	BDE (kJ/mol)
Chemical bond scission	ref. [10]	ref. [3]	ref. [44]
C=C	420		
C-C	335–350		
C-SC	300		298
C-SS	202		226
C-S_x_			226
CS-SC	277	285	290
CS-SSC		210	226
CSS-SSC	134	135	141
C-S_>4_-C			141

Furthermore, the presence of oxygen plays a key role in this NR molecular weight control, so that it is mechanochemical degradation (of the molecular weight). The experimental evidence that mechanical stress leads inevitably to the main chain chemical bond homolytic scission was demonstrated either by using free radical traps [2] or through a systematic and more convincing experimental approach using electron spin resonance (ESR), a spectroscopy specifically devoted to the detection and recognition of free radicals [47].

In the latest year polymer mechanochemistry has made incredible progresses [48,49,50] so that it is possible to use mechanochemistry in highly complex polymer structures making “chirurgical” changes along certain polymer chains and pendant groups. It is even possible to talk about mechanochemical synthesis of polymers [50] while previously mechanochemistry was seen essentially as a degradative technique. A particularly impressive concept developed in recent years is the concept of the “mechanophore”, a weak chemical bond purposely inserted into the polymer architecture (in general in the middle of the polymer backbone) to permit a selective breakdown of the polymer chains precisely on this weak bond where the stress forces concentrate [51]. It has also been found that in proteins, such sacrificial bonds which may be defined as mechanophore are just the disulfide bond as well as certain hydrogen bonds connecting intra- and intermolecularly certain protein units [52]. In other words, when certain large protein structures such as titin are subjected to external deformation forces, the dissipation of energy occurs by the breakdown of the mentioned sacrificial bonds (i.e., the disulfide crosslinks and certain hydrogen bonds) preventing the fragmentation of the main chain backbone [52]. It has been found also that upon stress release the broken sacrificial bonds may be reformed leading to a correct re-folding of the tertiary and quaternary protein structure [52]. 

**Scheme 2:**
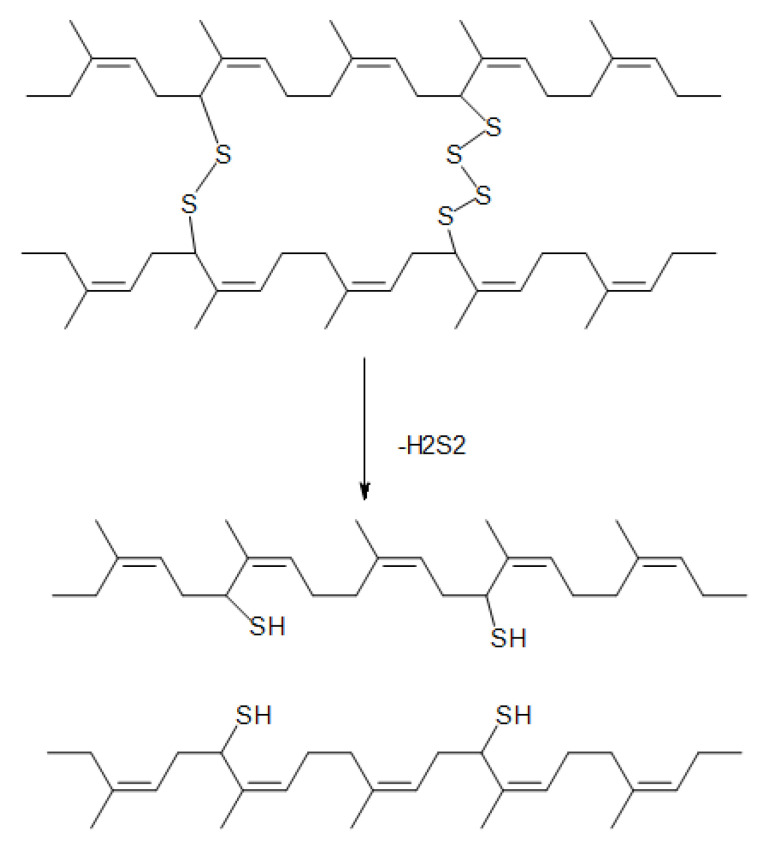
A simplified view of the devulcanization.

The analogy with rubber recycling is simply striking (*mutatis mutandis*); in fact, in cured rubber compounds we also have polysulfidic and disulfidic crosslinks which are characterized by very low and low BDE in comparison to the rubber chain backbone bonds C=C and C-C as shown in Table 6. Indeed, polysufidic and disulfidic bonds can be defined as “mechanophores” in a cured rubber network and hence could be considered as sacrificial bonds once the cured rubber network is submitted to the mechanical stress of external forces. It is however necessary to underline that only through a judicious administration of mechanical energy it could be possible to break selectively the sulfur crosslinks leaving intact the rubber chain backbone. Once this condition is fulfilled, we achieve the devulcanization condition which is depicted schematically in Scheme 2. Only the polysulfide, disulfide, and eventually the C-S bond of the polysulfidic crosslinks are broken down selectively by the external mechanical stress by bond homolysis leading to the formation of sulfur-centered free radicals. To avoid the possible recombination of the just mentioned S-centered radicals, chemical additives are used during the devulcanization process in order to stabilize provisory the devulcanized network. This specific aspect is treated in various reviews [25,26,27,28,29,30,31,32,33,34,35]. In Scheme 2 we show that the sulfur radicals derived by the mechanochemically induced homolysis are saturated by hydrogen atoms. Thus, truly devulcanized rubber can be re-vulcanized by a thermal treatment which implies for instance a very weak oxidation of the R-S_x_-H bonds back to the R-S_x_-S_x_-R or a recombination of the R-S_x_• radicals. The re-vulcanization of devulcanized rubber is still an exothermal process as the normal vulcanization process, although this time the heat released is expected to be lower to that released by a normal vulcanization since the devulcanization in general may not be 100% effective.

**Figure 7:**
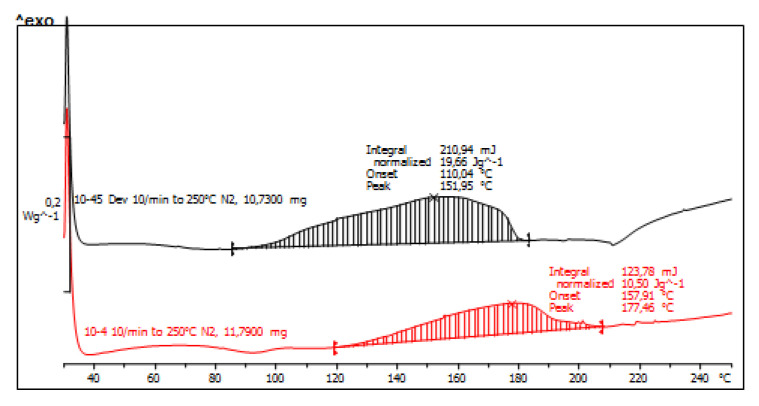
DSC analysis of two truly devulcanized rubber compounds showing the respective re-vulcanization exotherms.

This reasoning paves the way to a practical approach to identify a devulcanized rubber compound and even estimate approximately its degree of devulcanization. As shown in Figure 7, two truly devulcanized rubber compound were analyzed in a DSC at a heating rate of 10 °C/min. In both cases a broad exotherm attributed to re-vulcanization was detected. In one case the re-vulcanization peak occurred at 152 °C with an exotherm of 19.7 J/g, while in the second case the vulcanization peak was found at higher temperatures (i.e., 177 °C with an exotherm of 10.5 J/g). Based on these results, it is obvious that the first sample with the larger exotherm reached a higher degree of devulcanization than the second sample. 

**Figure 8:**
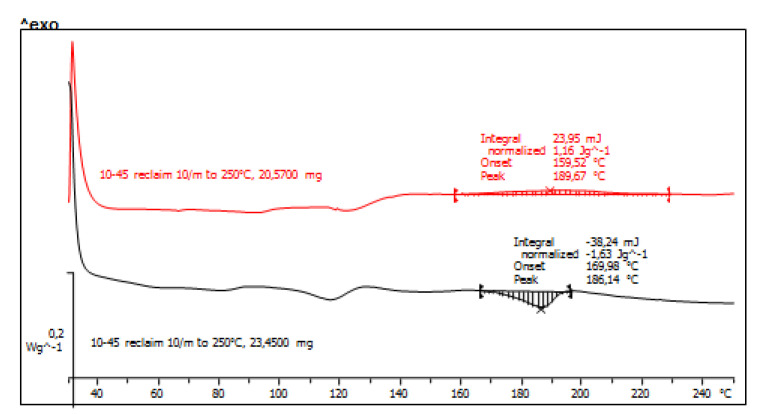
DSC analysis of two reclaimed rubber compounds which do not show any clear evidences of re-vulcanization exotherms.

On the other hand, the conventional reclaiming process of the rubber compound, which has been a consolidated industrial practice since the first half of the previous century, is based on the indiscriminate administration of mechanical energy to the cured rubber compound. The amount of energy administered is so large that inevitably there is a complete oxidative breakdown of the rubber chains backbone together with the sulfur crosslinks. The reclaimed compound obtained is necessarily much softer than the starting recycled compound (also as a result of the use of softeners and processing aids) and once tested in a DSC analysis as shown in Figure 8, it does not give any exothermal signal interpretable as a re-vulcanization process. In fact, in Figure 8 a reclaim sample gives at DSC just a very weak exotherm of only 1.2 J/g with peak shifted at 190 °C, while the other reclaim gives only an endothermal peak at 186 °C (probably due to the melting of certain processing aids used for the reclaim process).

Another feature of the devulcanized rubber with respect to the reclaimed rubber is the behavior in new rubber compounds. The devulcanized rubber can be used at much higher loadings than reclaimed rubber in new rubber compounds with minimal depression of the physical properties intended as moduli, elongation at break, and tensile strength. On the other hand, rubber reclaim can be used only in limited amounts in new rubber compounds. Due to the extensive mechanochemical degradation it has undergone, the reclaim inevitably depresses considerably the physical properties of a new rubber compounds limiting its re-utilization. In contrast, the devulcanized rubber permits a higher degree of recycling and re-utilization of recovered rubber compounds from used tires permitting a more effective implementation of the so-called circular economy in the rubber industry.

## 7. Experimental Section

### 7.1. Materials

The natural rubber used in this study was a TSR-20 grade. All of the other compounding ingredients used for the preparation of the rubber compounds (namely carbon black N375, mild extract solvate (MES) plasticizer, zinc oxide, stearic acid, trimethylquinoline (TMQ) antioxidant, paraffin wax, N-t-butyl-2-benzothyazyl sulphenamide (TBBS) accelerator and elemental sulfur) were all standard rubber industry grades. 

Two samples of devulcanized rubber were supplied by a primary Italian start-up company producing devulcanized rubber. 

Two rubber reclaim samples were standard rubber industry grades prepared according to conventional reclaiming processes of cured rubber [16].

### 7.2. Equipment

The differential scanning calorimeter used in this study was the Mettler-Toledo DSC-1 Star System. The calibration of the Mettler system is made regularly at least monthly using indium as conventional standard.

Mixing of the rubber compound formulations reported in Table 1 was made in a 2 L closed lab mixer from Farrel. The rheometer to control the kinetics of vulcanization of the rubber compounds was an ODR-type (Oscillating Disk Rheometer) from Gibitre Instruments.

### 7.3. Rubber Compound Mixing

Each rubber compound from A to E reported in Table 1 was mixed in two stages. In the first stage all of the compound ingredients were mixed together with the exclusion of TBBS accelerator and sulfur. After dumping, sheeting in an open mill, and cooling the masterbatch from the first stage was loaded again into the mixer and accelerator and sulfur were added, incorporated completely into the rubber compound, dumped, sheeted in an open mill, and cooled to room temperature. All compounds were checked with rheometer curve at 175 °C and 8 min. In Figure 2, some data derived from such measurement (i.e., the maximum torque and the delta torque) are reported.

### 7.4. DSC Measurements

#### 7.4.1. Measurement of the Vulcanization Heat in Al Crucibles

A weighed amount of each rubber compound (5–10 mg) of Table 1 was sealed in a conventional Al crucible (cap without punch) and heated at 10 °C/min under N_2_ flow from 25 °C to 350 °C in the DSC. The measured vulcanization exotherm was integrated using the Mettler software. It turned out as commented also in the appropriate section of this paper that the measurement in sealed Al crucible show a tendency to give a systematically lower vulcanization enthalpy than that reported in the literature [12,13,14,15]. 

#### 7.4.2. Measurement of the Vulcanization Heat in Medium Pressure Sealed Steel Crucibles

A weighed amount of each rubber compound (≥20 mg) of Table 1 was sealed (with O-ring inside) in a 30 μL stainless steel medium pressure crucibles from Mettler-Toledo (code 51140404) and heated at 20 °C/min under N_2_ flow from 25 °C to 350 °C in the DSC. The measured vulcanization exotherm was integrated using the Mettler software. It turned out that measurements with medium pressure crucibles yield vulcanization enthalpy values completely in line with literature data [12,13,14,15]. The uncertainty in the enthalpy of the vulcanization could be considered in the range of ±12%.

#### 7.4.3. Determination of the Activation Energy for the Vulcanization

The compounds B and E of Table 1 were heated in the DSC from 25 °C to 350 °C at different heating rates (e.g., 5 °C/min, 10°/min, 20 °C/min, 40°/min and 60 °C/min) in sealed medium pressure crucibles. The data from the two series of measurements were treated according to the Ozawa and Kissinger equations [42] and the activation energy was determined for each compound.

#### 7.4.4. DSC Study of Re-Vulcanization of Devulcanized Rubber or Rubber Reclaim

A weighed amount of devulcanized rubber or reclaimed rubber was sealed in medium pressure crucible and heated in the DSC at 10 °C/min from 25 to 250 °C under N_2_ flow. The resulting DSC trace showed an exothermal transition in the case of the two devulcanized rubber samples (Figure 7) which were revulcanized by heating. Instead, the rubber reclaim samples show a flat DSC curve with no exotherms (Figure 8).

#### 7.4.5. Devulcanization Process

The devulcanization process adopted is based on the patent [53] and some details can be found also in the ref. [25], where the patent [53] is cited.

## 8. Conclusions

Since the vulcanization is an exothermal process, then also truly devulcanized rubber once re-vulcanized should show an exothermal transition due to re-vulcanization at the DSC scan. In contrast, common and conventional reclaimed rubber does not show any exothermal transition at the DSC.

To demonstrate this thesis, first of all a standard NR-based and carbon black filled compound was mixed with different levels of sulfur as shown in Table 1. The vulcanization of these compounds was studied with a DSC. It was found that Al crucibles are not the most suitable crucibles for this study and that it is better to work with steel, medium pressure crucibles. It was found that the vulcanization enthalpy is dependent on the amount of sulfur present in the compound and the vulcanization heat released was −18.4 kJ/mol S if referred to the entire rubber compound formulation or −46.0 kJ/mol S if the heat released is referred only to the NR present in the compound. These conclusions are in good agreement with the results obtained by the scientists of the NBS [12,13,14,15]. A simplified model for the chemical explanation of the vulcanization has been presented and used for a simplified thermochemical calculation of the vulcanization (Scheme 1). The model correctly predicts the exothermicity of the vulcanization process although it tends to overestimate the vulcanization heat with respect to the experimental value. The overestimation is larger for the compounds with EV cure package and is greatly reduced for the compounds with conventional cure system. This is due to the fact that the proposed simplified model for the vulcanization is essentially based on the unaccelerated sulfur-based vulcanization, but the results of the model are compared with the experimental results of the accelerated rubber compound formulation of Table 1.

Furthermore, the activation energy for the vulcanization of the rubber compounds in Table 1 was also determined by a DSC study at 49 kJ/mol and found to be quite independent from the sulfur content of the compounds under study. An explanation was given to the reason why the experimental activation energy is about 1/3 the value theoretically expected for unaccelerated rubber compound vulcanization: it is due the effect of the accelerator and the activators (ZnO and stearic acid).

The proposed simplified model of vulcanization is also useful for explaining in a simplified way the process of devulcanization (Scheme 2). If the mechanochemical energy is administered selectively to the cured rubber compound (for instance recovered from used tires) in order to break only the mechanophore bonds then a truly devulcanization process is achieved. The mechanophore bonds in a cured rubber network are just the weaker bonds in terms of BDE as shown in Table 6 (i.e., for instance the polysulfidic, disulfidic as well as the C-S bonds in polysulfidic bridges). By judicious administration of mechanochemical energy, the above-mentioned bonds are broken essentially by homolysis producing free radicals which are stabilized by the addition of additives. The resulting devulcanized rubber, once analyzed in a DSC, re-vulcanizes, producing again an exothermal transition as shown for instance in Figure 7. This analytical approach permits one to distinguish truly devulcanized rubber from common reclaimed rubber which instead when analyzed with a DSC does not show any exotherm (Figure 8). Reclaimed rubber is produced as well from cured rubber recovered for instance from used tires but with an indiscriminate administration of mechanochemical energy so that the degradation due to bond scission involves any chemical bond present in the rubber network including the rubber C-C bond. This leads to a complete degradation of the rubber network and the resulting product, loaded also by softeners and other additives, cannot be defined at all “devulcanized” but rather a conventional “reclaimed rubber” (another product different from truly “devulcanized rubber”).

Of couse, the practical difference between devulcanized and reclaimed rubber is that the former can be recycled at much higher loadings in new rubber compound formulations than the latter, without significant depression of the mechanical properties of the resulting compound.

## Figures and Tables

**Table 1:** NR-based compound formulation with variable levels of sulfur.

	A	B	C	D	E
	phr	phr	phr	phr	phr
Natural Rubber (TSR-20)	100	100	100	100	100
Carbon black N375	50	50	50	50	50
MES OIL plasticizer	5	5	5	5	5
Zinc oxide	5	5	5	5	5
Stearic acid	3	3	3	3	3
TMQ	1	1	1	1	1
WAX	1	1	1	1	1
TBBS	1.5	1.5	1.5	1.5	1.5
**SULPHUR**	**0.5**	**1**	**2**	**4**	**6**
total phr	167.0	167.5	168.5	170.5	172.5

Notes: zinc oxide and stearic acid are cure activators; TMQ is trimethylquinoline-based antioxidant; WAX is a paraffin and it is a static antiozonant; TBBS is ter-butylbenzothiazyl sulphenamide and it is a common accelerator. Sulfur level is shown in bold to outline the changes in each formulation.

**Table 2:** Vulcanization enthalpy of NR-rubber compounds of reported in Table 1 heating rate 10 °C/min in sealed Al crucible.

phr of Sulfur	Onset °C	Peak °C	−ΔH_vulc_ (J/g)
0.5	144.8	179.2	2.7
1.0	167.5	182.1	4.2
2.0	165.6	179.2	7.6
4.0	170.0	179.7	15.3
6.0	185.7	202.6	22.3

**Table 3:** Vulcanization Enthalpy of NR-rubber compounds of reported in Table 1 heating rate 20 °C/min in sealed medium pressure stainless steel crucibles.

phr of Sulfur	Onset °C	Peak °C	−ΔH_vulc_ (J/g)
0.5	175.3	203.1	1.16
1.0	186.7	205.3	3.12
2.0	181.7	198.0	11.8
4.0	180.2	205.0	20.9
6.0	178.3	208.1	30.0

**Table 4:** Enthalpy of reaction cyclooctasulfur ring scission into radicals.

Reaction Considered	ΔH_r_ (kJ/mol) (*)	ΔH_r_ (kJ/mol) (**)
S_8_ -> 8 S	2230	2220
S_8_ -> 4 S_2_	514	514
S_8_ -> 2 S_3_ + S_2_	394	401
S_8_ -> S_7_ + S		381
S_8_ -> 8/3 S_3_	355	363
S_8_ -> 2 S_4_	274	260
S_8_ -> S_5_ + S_3_	257	
S_8_ -> S_6_ + S_2_		231
S_8_ -> 8/5 S_5_	198	198
S_8_ -> 4/3 S_6_	165	137
S_8_ -> 8/7 S_7_	142	117
_ring_-S_8_ -> _chain_-S_8_	137–150 (***)	137

(*) calculations using enthalpies of formation from ref. [43]. (**) calculations using enthalpies of formation from ref. [44]. (***) data reported in the review [36].

**Table 5:** Enthalpy of reaction for the formation of the sulfur crosslinks between rubber adjacent chains.

Crosslink Nature	Monosulfide Bridge	Disulfide Bridge	Trisulfide Bridge	Tetrasulfide Bridge
Enthalpy of Reaction	ΔH_r_ (kJ/mol)	ΔH_r_ (kJ/mol)	ΔH_r_ (kJ/mol)	ΔH_r_ (kJ/mol)
Reaction A	−45.8	57.6	69.0	78.0
Reaction B	−65.6	17.5	17.0	17.3
Reaction C–D	−408.8	−216.3	−201.9	−189.2
Sum of all reaction A + B + C/D enthalpy (kJ/mol)	−520.2	−141.2	−115.9	−93.9
